# CAIDE dementia risk score relates to severity and progression of cerebral small vessel disease in healthy midlife adults: the PREVENT-Dementia study

**DOI:** 10.1136/jnnp-2021-327462

**Published:** 2022-02-08

**Authors:** Audrey Low, Maria A Prats-Sedano, James D Stefaniak, Elizabeth Frances McKiernan, Stephen F Carter, Maria-Eleni Douvani, Elijah Mak, Li Su, Olivia Stupart, Graciela Muniz, Karen Ritchie, Craig W Ritchie, Hugh S Markus, John Tiernan O'Brien

**Affiliations:** 1 Department of Psychiatry, School of Clinical Medicine, University of Cambridge, Cambridge, UK; 2 Division of Neuroscience and Experimental Psychology, The University of Manchester, Manchester, UK; 3 Department of Clinical Neurosciences, School of Clinical Medicine, University of Cambridge, Cambridge, UK; 4 Department of Neuroscience, The University of Sheffield, Sheffield, UK; 5 Centre for Dementia Prevention, University of Edinburgh, Edinburgh, UK; 6 INSERM, Montpellier, France

**Keywords:** dementia, cerebrovascular disease

## Abstract

**Background:**

Markers of cerebrovascular disease are common in dementia, and may be present before dementia onset. However, their clinical relevance in midlife adults at risk of future dementia remains unclear. We investigated whether the Cardiovascular Risk Factors, Ageing and Dementia (CAIDE) risk score was associated with markers of cerebral small vessel disease (SVD), and if it predicted future progression of SVD. We also determined its relationship to systemic inflammation, which has been additionally implicated in dementia and SVD.

**Methods:**

Cognitively healthy midlife participants were assessed at baseline (n=185) and 2-year follow-up (n=158). To assess SVD, we quantified white matter hyperintensities (WMH), enlarged perivascular spaces (EPVS), microbleeds and lacunes. We derived composite scores of SVD burden, and subtypes of hypertensive arteriopathy and cerebral amyloid angiopathy. Inflammation was quantified using serum C-reactive protein (CRP) and fibrinogen.

**Results:**

At baseline, higher CAIDE scores were associated with all markers of SVD and inflammation. Longitudinally, CAIDE scores predicted greater total (p<0.001), periventricular (p<0.001) and deep (p=0.012) WMH progression, and increased CRP (p=0.017). Assessment of individual CAIDE components suggested that markers were driven by different risk factors (WMH/EPVS: age/hypertension, lacunes/deep microbleeds: hypertension/obesity). Interaction analyses demonstrated that higher CAIDE scores amplified the effect of age on SVD, and the effect of WMH on poorer memory.

**Conclusion:**

Higher CAIDE scores, indicating greater risk of dementia, predicts future progression of both WMH and systemic inflammation. Findings highlight the CAIDE score’s potential as both a prognostic and predictive marker in the context of cerebrovascular disease, identifying at-risk individuals who might benefit most from managing modifiable risk.

## Introduction

Cerebral small vessel disease (SVD) is recognised as a major contributor in the pathogenesis of neurodegeneration, including Alzheimer’s disease (AD).^1 2^ SVD affects perforating vessels in the brain and is closely associated with modifiable vascular risk factors, such as high blood pressure, smoking and obesity. Importantly, such risk factors are more predictive of cognitive decline at midlife, and lose predictive value in older age.^3–6^ Taken together, the prevailing evidence highlights opportunities for preventative interventions, particularly during midlife. Systemic inflammation is also being increasingly implicated in cerebrovascular health and neurodegeneration.^1 7–9^ Although it normally serves a protective function in response to infections and injuries, a prolonged inflammatory response can be detrimental to surrounding tissue. Inflammation has been suggested to be both a risk factor for SVD, and a possible pathogenic mechanism mediating the effect of vascular risk factors on the small cerebral arteries.^7^

Emerging evidence suggest that cerebrovascular alterations constitute the early preclinical phases of incipient dementia.^10^ This highlights the importance of understanding the risk factors and implications of early vascular changes to shed light on early mechanisms in dementia and facilitate early detection and disease management, which this present study aimed to address by investigating cognitively asymptomatic middle-aged adults at risk of developing dementia.

The importance of early detection in the management of neurodegenerative diseases has given rise to the development of dementia risk scores, such as the Cardiovascular Risk Factors, Ageing and Dementia (CAIDE) score. Developed in middle-aged community subjects, the CAIDE risk score is calculated based on age, sex, education, systolic blood pressure, body mass index (BMI), total cholesterol, physical activity and apolipoprotein ε4 (APOE4), and has been validated to estimate the risk of dementia 20 years later.^11^ Furthermore, the CAIDE score has also been shown to predict longitudinal brain atrophy even at midlife.^12^ The limited literature on CAIDE associations with SVD suggests that higher CAIDE scores relate to greater white matter hyperintensities (WMH), but not cerebral microbleeds (CMB) in cross-sectional analyses, although investigation on longitudinal SVD progression has been lacking.^13 14^ Furthermore, the CAIDE score has not been assessed in relation to other key SVD markers like enlarged perivascular spaces (EPVS), lacunes, composite SVD measures or with any markers of inflammation, cross-sectionally or longitudinally.

Given the close association between dementia and cerebrovascular health, coupled with the cardiovascular components of the CAIDE score (eg, blood pressure, cholesterol), we aimed to investigate the association between midlife CAIDE score with SVD and inflammation cross-sectionally, and importantly, whether it predicted progression longitudinally over 2 years at midlife. We hypothesised that higher CAIDE scores would relate to both greater baseline severity and increased longitudinal change in markers of SVD and systemic inflammation. Topographically, we hypothesised that associations would be more pronounced for SVD lesions in regions typically affected by hypertensive arteriopathy (eg, basal ganglia) as opposed to cerebral amyloid angiopathy (CAA-SVD) (eg, lobar CMB), since the former involves vascular injury to the deep perforating arteries susceptible to hypertension-related alterations, while the latter relates to the deposition of amyloid-β within vessel walls.^15–18^

## Methods

### Participants

The protocol of the PREVENT-Dementia study has been described in detail previously.^19^ Cognitively healthy, midlife participants aged 40–59 years were recruited; details in online supplemental materials. The study recruited 210 participants at baseline, of which 193 had an MRI scan at baseline. Of the 193, 6 were excluded due to incidental findings (5 meningiomas, 1 brain tumour) and 2 were excluded due to missing APOE data, leaving 185 participants with full baseline data, of which 158 returned at follow-up.

10.1136/jnnp-2021-327462.supp1 Supplementary data

### CAIDE dementia risk score

CAIDE scores were calculated for each participant at baseline.^11^ The CAIDE score is composed of weightings by reference to age, sex, education, systolic blood pressure, BMI, total cholesterol, physical activity and APOE4 genotype (all variables measured at baseline). The CAIDE score ranges from 0 to 18, whereby a higher score represents higher risk of future dementia. For the specific purposes of data visualisation and confirmation of interaction results only, we binarized the CAIDE score using a cut-off of ≥6, following the recommendation of its developers who adopted this cut-off in the Finnish Geriatric Intervention Study to Prevent Cognitive Impairment and Disability (FINGER) study.^20^ The raw CAIDE score (range 0–18) was used for all other analyses and purposes.

### Imaging

MRI scans were acquired at baseline and 2-year follow-up on a 3 T Siemens Verio; acquisition parameters detailed in online supplemental materials.

### Quantification of SVD

#### Semi-quantitative measurements

**Figure 1:**
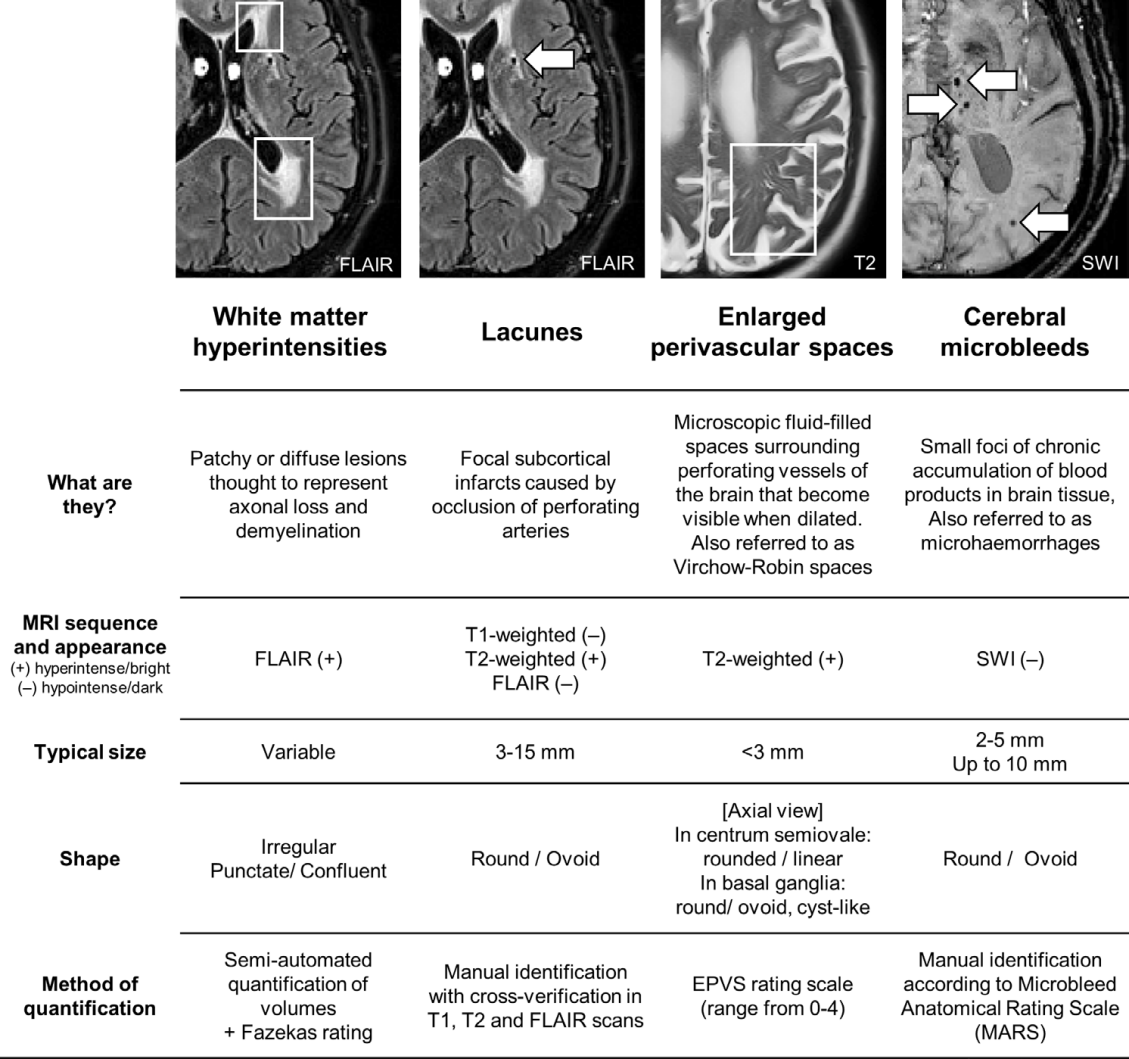
Imaging markers of cerebral small vessel disease. EPVS, enlarged perivascular spaces; FLAIR, fluid-attenuated inversion recovery; SWI, susceptibility-weighted imaging.

WMH were visually rated on fluid-attenuated inversion recovery (FLAIR) MRI according to the Fazekas scale for the computation of composite SVD scores.^21^ EPVS were rated on T2-weighted MRI using a validated rating scale,^22^ and were assessed separately in the basal ganglia and centrum semiovale to account for different underlying pathologies. EPVS scores ranged from 0 to 4 according to number of EPVS: 0 (none), 1 (1–10), 2 (11–20), 3 (21–40), 4 (>40). Lacunes were identified using T1-weighted, T2-weighted and FLAIR scans, following the STandards for ReportIng Vascular changes on nEuroimaging (STRIVE) guidelines.^23^ CMB were identified on susceptibility-weighted imaging (SWI) following the Microbleed Anatomical Rating Scale.^24^ Visual rating procedures are described in detail under online supplemental materials and figure 1. Each SVD marker was rated by a single rater, and 20% were rated by a second rater. Raters were blinded to all clinical measures, and inter-rater reliability (Cohen’s kappa) were as follows: CMB: 0.92, lacunes: 0.89, EPVS: 0.93 in centrum semiovale, 0.93 in basal ganglia, WMH: 0.85 for periventricular, 0.73 for deep. All discrepancies and uncertain cases were discussed, and consensus was met among raters.

### Quantitative measure of WMH volume

**Figure 2:**
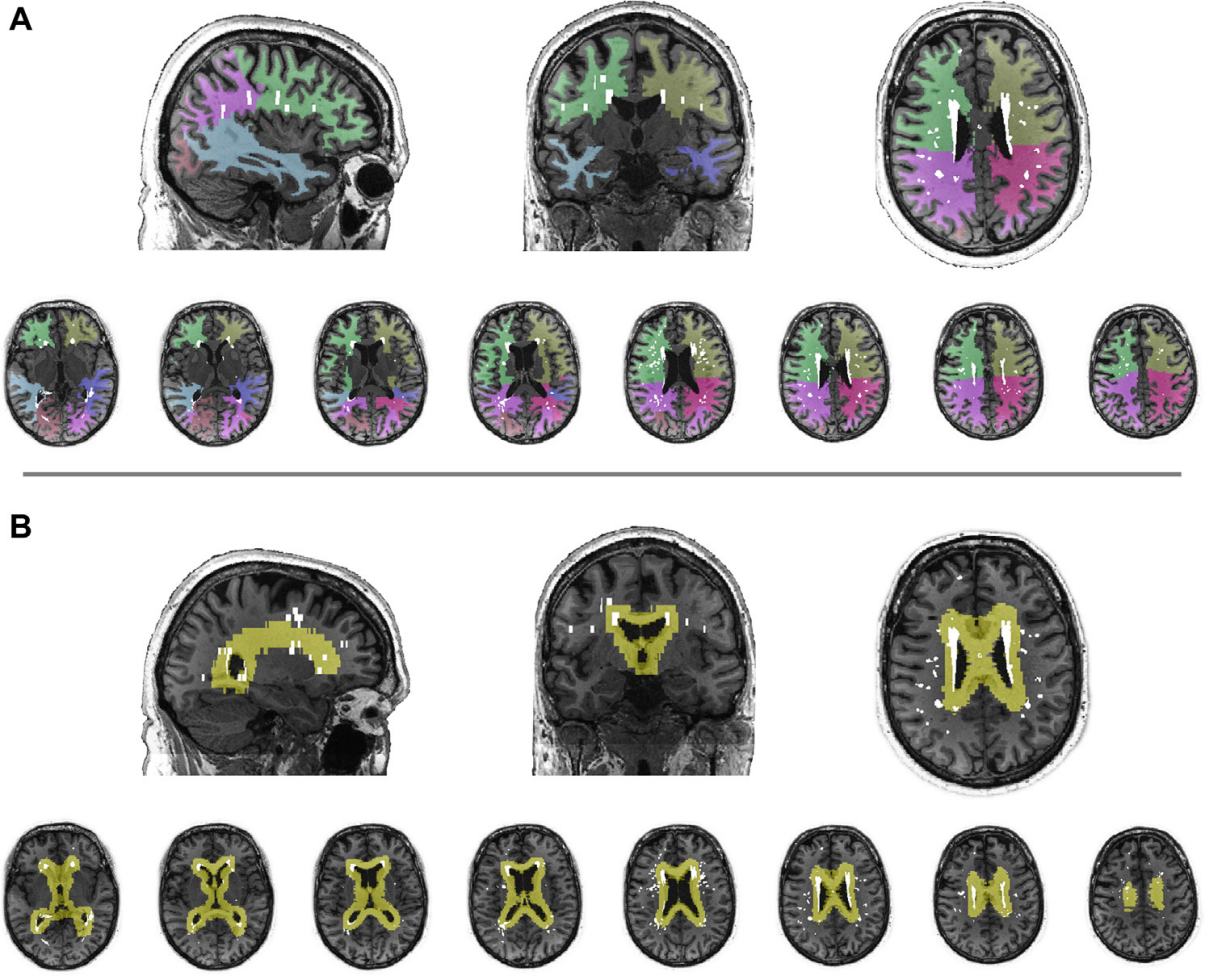
Topographical segmentation of white matter hyperintensity (WMH) burden. WMH volumes were quantified into (A) lobar regions (frontal, temporal, parietal, occipital) and (B) periventricular and deep WMH.

WMH lesion maps were obtained using an automated script on the Statistical Parametric Mapping 8 suite (http://www.fil.ion.ucl.ac.uk/spm/) on FLAIR MRI; details described elsewhere,^25^ and under online supplemental materials. WMH were segmented into lobar regions (frontal, parietal, occipital, temporal) using a region of interest template, as previously described.^25^ WMH were also classified as periventricular or deep WMH based on threshold distance from ventricles (figure 2). WMH volumes were normalised by total intracranial volume (TIV) to account for individual differences in head size ((WMH/TIV)×100%) and underwent cube root transformation due to right-tailed skewness.

### Composite SVD scores

Composite SVD burden scores were computed using a point system, based on the presence or absence of each of the four SVD markers, according to cut-offs defined in earlier studies.^26 27^ Global SVD burden scores were computed according to Staals *et al*,^26^ while the two SVD subtypes of CAA-SVD and hypertensive arteriopathy were formulated according to distinctions made in the literature,^16–18 28 29^ assigning one point per criterion met. For CAA-SVD, one point was awarded for each criterion: (1) one or more lobar lacunes, (2) one or more lobar CMB, (3) EPVS score in centrum semiovale ≥2, (4) periventricular WMH Fazekas=3 and/or deep WMH Fazekas ≥2. Similarly, for hypertensive arteriopathy: (1) one or more deep lacunes, (2) one or more deep CMB, (3) EPVS score in basal ganglia ≥2, (4) deep WMH Fazekas ≥2.

### Measures of systemic inflammation

Inflammation was quantified using two widely studied serum markers of systemic inflammation, C-reactive protein (CRP) and fibrinogen.^30^ CRP was analysed on the Beckman Coulter AU System using a CRP latex reagent (Beckman Coulter, Brea, California, USA). As a form of sensitivity analysis, we analysed CRP data both with and without exclusion of participants with CRP exceeding 10 mg/L in separate analyses to examine the consistency of results. Core analysis was conducted with the full sample (ie, represented in tables and figures), while filtered data were used in secondary analyses. Fibrinogen was measured using the Clauss method.^31^ CRP and fibrinogen data underwent cube root transformation due to skewness.

### Genotyping

TaqMan genotyping on QuantStudio12K Flex was used to establish APOE variants; details in online supplemental materials.

### Neuropsychological measures

Participants underwent neuropsychological assessment at baseline and 2-year follow-up. As part of the COGNITO battery,^32^ participants were assessed on reaction time, executive function (Stroop test) and episodic memory (recall task); details under online supplemental materials.

### Statistical analysis

All statistical analyses were conducted using R (v3.6.3; www.R-project.org).

To test associations between the CAIDE score and baseline severity of SVD and systemic inflammation, Spearman’s rank correlation was used. To further assess the relative contribution of individual CAIDE components (eg, age, hypertension, obesity) to each marker of SVD and inflammation, general linear models were fitted to each biomarker in separate models, with all CAIDE components included as predictors (results under ‘Baseline associations’ section).

To assess whether baseline CAIDE scores were associated with *longitudinal* changes of SVD markers and inflammation, linear mixed-effects models were fitted using the *lme4* package in R to assess changes in WMH, EPVS, CRP and fibrinogen, with *CAIDE×time* interaction terms as fixed effects, and subject intercept as random effects. Spearman’s correlations were used to analyse CAIDE associations with the presence/absence of new CMB/lacunes at follow-up. To assess the relative contribution of individual CAIDE components to longitudinal changes of SVD and systemic inflammation, linear fixed-effects models were fitted to each SVD/inflammatory marker in separate models, entering all CAIDE components as predictors as *CAIDE component×time* interaction terms (results under ‘Longitudinal associations’ section).

To assess the effects of age on SVD and inflammation, univariate linear mixed-effects models were fitted to SVD and inflammation markers in separate models. To evaluate whether CAIDE scores moderated these relationships, *age at visit×baseline CAIDE* interaction terms were added to each linear mixed-effects model in a subsequent step. To validate significant interactions, raw CAIDE scores were binarized using a cut-off of CAIDE ≥6, and general linear models were fitted separately in the CAIDE <6 and CAIDE ≥6 groups, with age as the predictor and SVD as the outcome (results under ‘Interaction of CAIDE and age’ section).

To examine CAIDE associations with cognitive measures and SVD markers, Spearman’s correlations were conducted to test for baseline associations, while linear mixed-effects models were fitted to assess longitudinal associations. To investigate potential *CAIDE×SVD* interactions on cognition, interaction analysis was conducted using general linear models for baseline interactions and linear mixed-effects models for longitudinal interactions (results under ‘Cognition’ section).

False discovery rate (FDR) correction was applied for multiple comparisons across all markers.^33^ Statistical significance was set at FDR-adjusted p<0.05.

## Results

The sample was majority female (69.7%), had a mean age of 51.9 years (SD=5.4) at baseline and an average of 15.9 years of education (SD=3.4) (table 1). Mean interval between baseline and follow-up was 2.0 years (SD=0.2).

**Table 1:** Participant characteristics

N		Baseline	Follow-up
		185	158
Demographics			
Sex	% females	69.7%	69.0%
Age in years	Mean (SD)	51.9 (5.5)	54.2 (5.4)
Education in years	Mean (SD)	15.9 (3.4)	16.1 (3.4)
CAIDE score (range 0–18)	Mean (SD)	5.8 (2.9)	5.9 (2.9)
APOE4	% carriers	37.3%	38.0%
APOE2	% carriers	9.7%	8.2%
Hypertension	% positive	14.1%	15.1%
Hyperlipidaemia	% positive	17.3%	19.2%
Diabetes mellitus	% positive	3.2%	3.5%
Medication			
Antihypertensive medication	% on medication	7.6%	10.5%
Antihyperlipidaemic medication	% on medication	4.3%	7.6%
Antidiabetic medication	% on medication	1.6%	2.3%
SVD markers			
WMH volume (% of TIV)	Mean (SD)	0.10 (0.14)	0.12 (0.19)
Lacunes (present/absent)	% present	10.8%	12.0%
Cerebral microbleeds (present/absent)	% present	18.4%	24.0%
Enlarged perivascular spaces (range 0–4)	Mean (SD)	0.72 (0.54)	0.72 (0.55)
Composite SVD scores			
Global SVD (range 0–4)	Mean (SD)	0.39 (0.65)	0.44 (0.66)
CAA-SVD (range 0–4)	Mean (SD)	0.45 (0.67)	0.47 (0.70)
Hypertensive arteriopathy (range 0–4)	Mean (SD)	0.18 (0.47)	0.20 (0.48)
Inflammatory markers			
C reactive protein (mg/L)	Mean (SD)	3.14 (2.72)	3.20 (3.98)
Fibrinogen (g/L)	Mean (SD)	3.05 (0.69)	3.03 (0.72)

All participants had complete baseline data; four did not have follow-up CMB data.

CAIDE, Cardiovascular Risk Factors, Aging and Dementia; SVD, small vessel disease; WMH, white matter hyperintensities; TIV, total intracranial volume; CAA-SVD, SVD subtype of cerebral amyloid angiopathy.

### Baseline associations

**Figure 3:**
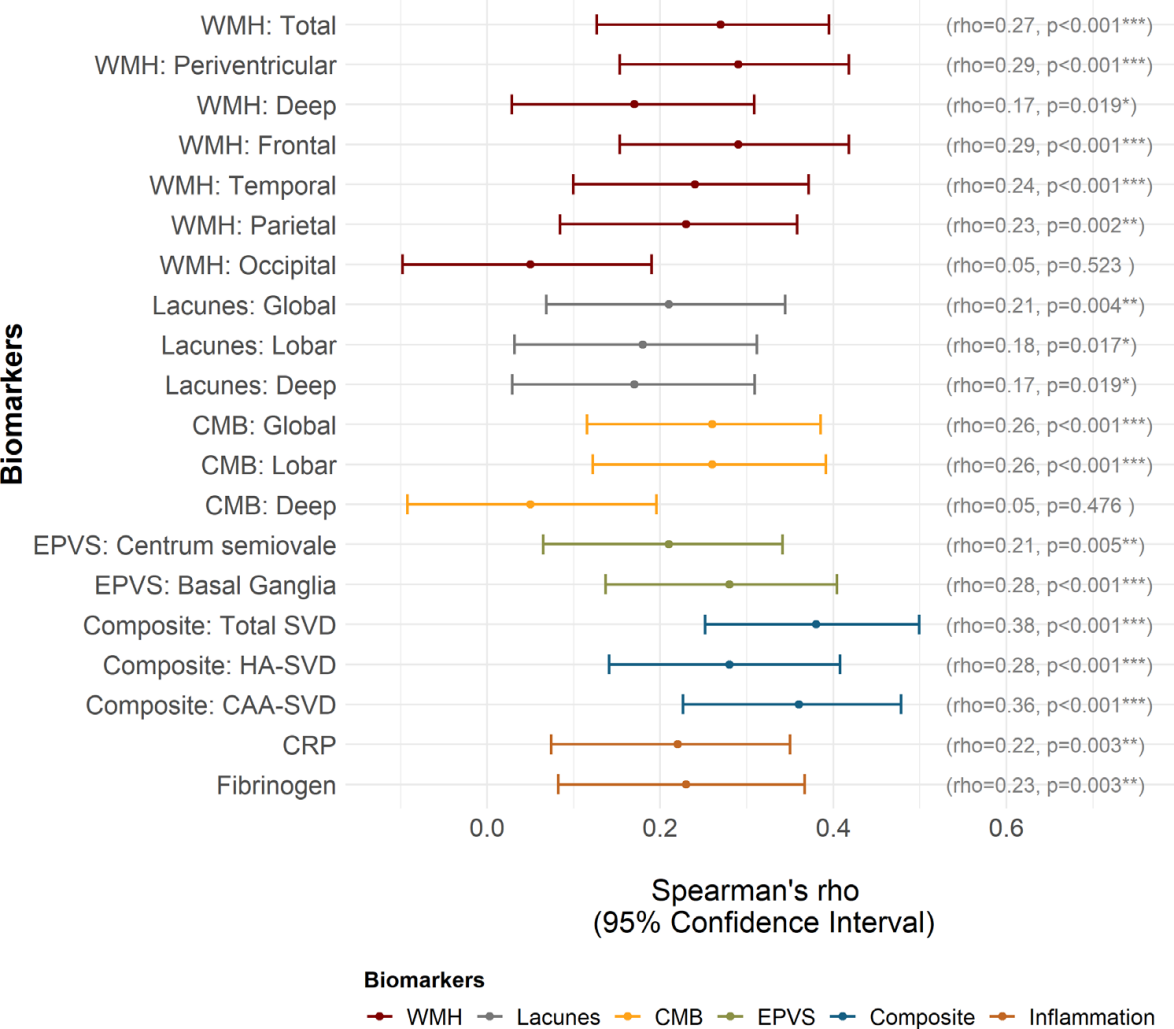
Strength of association between Cardiovascular Risk Factors, Ageing and Dementia CAIDE) score and each biomarker at baseline. P values are corrected for false discovery rate. *P<0.05, **p<0.01, ***p<0.001. CAA, cerebral amyloid angiopathy; CMB, cerebral microbleeds; CRP, C-reactive protein; EPVS, enlarged perivascular spaces; HA, hypertensive arteriopathy; SVD, small vessel disease; WMH, white matter hyperintensities.

Higher CAIDE scores were associated with greater WMH volume (both periventricular and deep), presence of lacunes (both lobar and deep), CMB (lobar, but not deep), EPVS (both centrum semiovale and basal ganglia) and total composite scores of global SVD, CAA-SVD and hypertensive arteriopathy. CAIDE scores were also associated with inflammation (CRP and fibrinogen) (figure 3). We performed additional sensitivity analyses to examine whether results changed when cases with markedly raised CRP (>10 mg/L) were excluded, and observed similar results (r_s=_0.24, p_FDR_=0.001).

**Figure 4:**
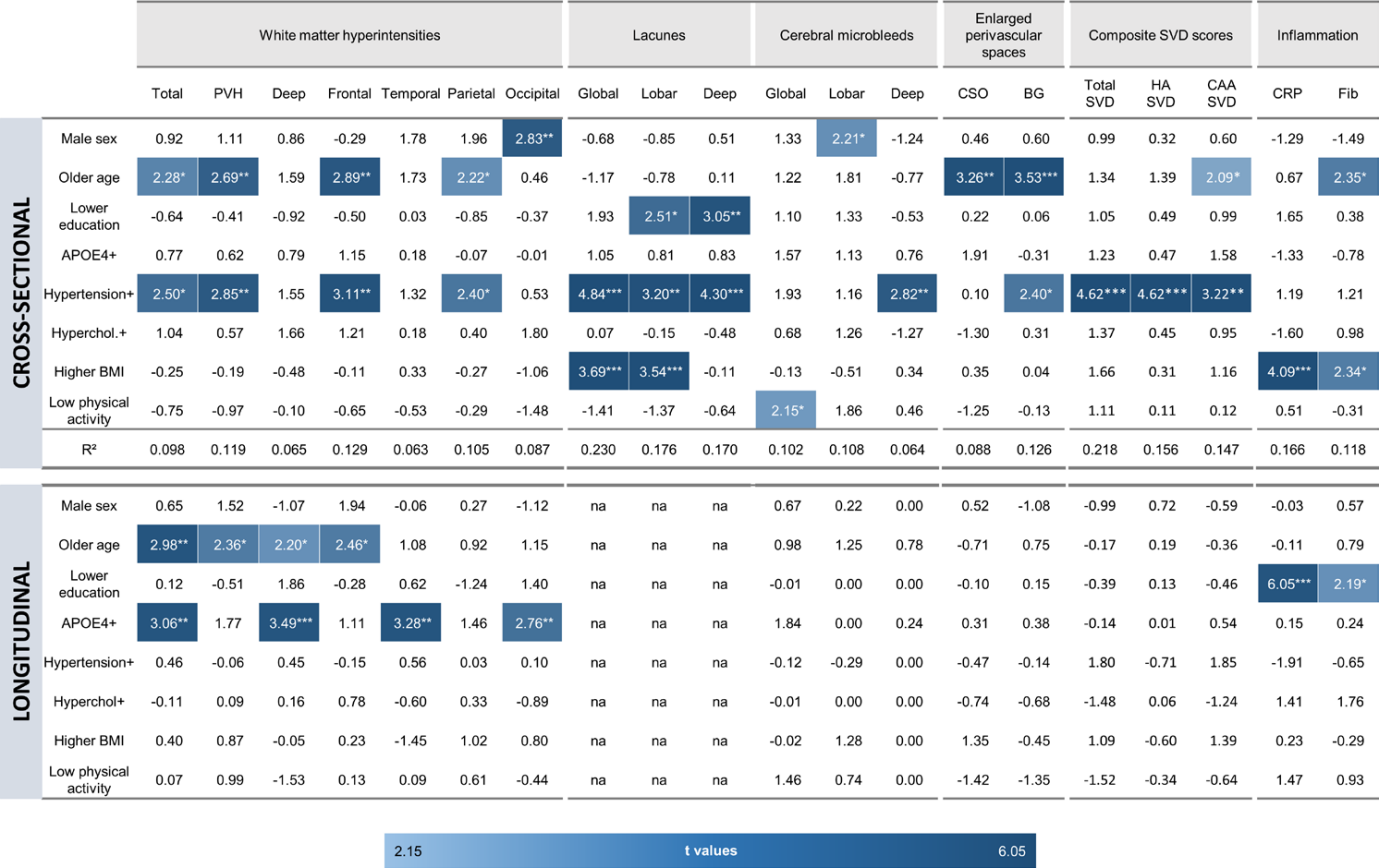
Heatmaps depicting the relative contribution of Cardiovascular Risk Factors, Ageing and Dementia components to each marker of SVD and inflammation. Top: baseline associations analysed using general linear modelling. Bottom: longitudinal associations analysed using linear mixed-effects modelling. Coloured cells represent statistically significant components (p<0.05), and darker shades indicate stronger associations. *P<0.05, **p<0.01, ***p<0.001. BMI, body mass index; BG, basal ganglia; CAA, cerebral amyloid angiopathy; CRP, C reactive protein; CSO, centrum semiovale; Fib, fibrinogen; HA, hypertensive arteriopathy; Hyperchol, hypercholesterolaemia; PVH, periventricular white matter hyperintensities; SVD, small vessel disease.

To assess the contribution of individual CAIDE components to SVD, general linear models were fitted, entering all CAIDE components as predictors (figure 4). Accounting for all other components, age and hypertension were the greatest contributors to SVD, although lacunes were most closely linked to lower educational attainment, hypertension and higher BMI. Hypertension was the strongest predictor of *deep* microbleeds, while male sex was the strongest predictor of *lobar* microbleeds. Hypertension was also the strongest predictor of all three composite SVD scores, while the strongest contributor to inflammation was high BMI (figure 4).

#### Longitudinal associations

Linear mixed-effects modelling demonstrated significant *CAIDE×time* interactions on WMH volumes (total, periventricular, deep, frontal, parietal), such that higher baseline CAIDE scores predicted greater future WMH progression (table 2). Baseline CAIDE scores did not predict the progression of CMB, EPVS, or composite SVD scores. Statistical testing of lacune progression was not conducted as only two participants (n=2; 1.27%) developed new lacunes at follow-up—both participants had CAIDE scores ≥6 and had strictly lobar lacunes at baseline; new lacunes were observed in the same region as existing lacunes. Baseline CAIDE scores were also related to greater increase in CRP levels (t=2.41, p_FDR_=0.049) (table 2).

**Table 2:** CAIDE score associations with progression of SVD markers

	Statistics	P value	FDR-corrected p value
**White matter hyperintensities**			
Total	t=3.44	<0.001***	0.004**
Periventricular	t=3.72	<0.001***	0.004**
Deep	t=2.55	0.012*	0.043*
Frontal	t=3.52	<0.001***	0.004**
Temporal	t=0.51	0.609	0.760
Parietal	t=2.52	0.013*	0.043*
Occipital	t=1.75	0.082	0.164
**Cerebral microbleeds**			
Whole brain	r_s_=0.14	0.087	0.164
Lobar	r_s_=0.13	0.113	0.193
Deep	r_s_=0.06	0.458	0.649
**Enlarged perivascular spaces**			
Centrum semiovale	t=−0.41	0.679	0.760
Basal ganglia	t=−0.37	0.715	0.760
**Composite SVD scores**			
Global SVD	t=0.49	0.625	0.759
HA-SVD	t=−0.07	0.947	0.947
CAA-SVD	t=0.95	0.344	0.531
**Inflammation**			
CRP	t=2.41	0.017*	0.049*
Fibrinogen	t=1.79	0.076	0.164

WMH and EPVS progression analysed using linear mixed-effects modelling; CMB progression analysed using Spearman’s correlation. Lacune progression not analysed due to low incidence of new lacunes.

*P<0.05, **p<0.01, ***p<0.001.

CAA, cerebral amyloid angiopathy; CAIDE, Cardiovascular Risk Factors, Ageing and Dementia; CMB, cerebral microbleeds; CRP, C reactive protein; EPVS, enlarged perivascular spaces; FDR, false discovery rate; HA, hypertensive arteriopathy; SVD, small vessel disease; WMH, white matter hyperintensities.

In terms of individual CAIDE components, linear mixed-effects models accounting for time interaction with all CAIDE components at baseline showed that age and APOE4 were the strongest predictors of WMH progression (figure 4). Adjusting for all other factors, older age was associated with greater WMH progression (total, periventricular, deep, frontal), while APOE4 was significantly related to the progression of total, deep, temporal and occipital WMH. No individual component remained significant in models fitted to the progression of CMB, EPVS or global SVD composite scores. For inflammation, lower education was the strongest predictor of increasing CRP and fibrinogen levels over 2 years.

### Interaction of CAIDE and age

#### Main effects

In linear mixed-effects models, older age related to greater WMH (total: t=3.32, p<0.001, periventricular: t=4.38, p<0.001, deep: t=1.98, p=0.049), EPVS (basal ganglia: t=5.57, p<0.001; centrum semiovale: t=4.31, p<0.001), presence of CMB globally (z=3.56, p<0.001) and in lobar regions (z=4.17, p<0.001), composite SVD measures (global SVD: t=4.14, p<0.001; CAA-SVD: t=4.46, p<0.001; hypertensive arteriopathy: t=2.91, p=0.004) and inflammation (CRP: t=2.02, p=0.044; fibrinogen: t=4.73, p<0.001). However, age was not associated with lacunes (z=1.31, p=0.190) or deep CMB (z=0.32, p=0.751).

#### Interaction effects

**Figure 5:**
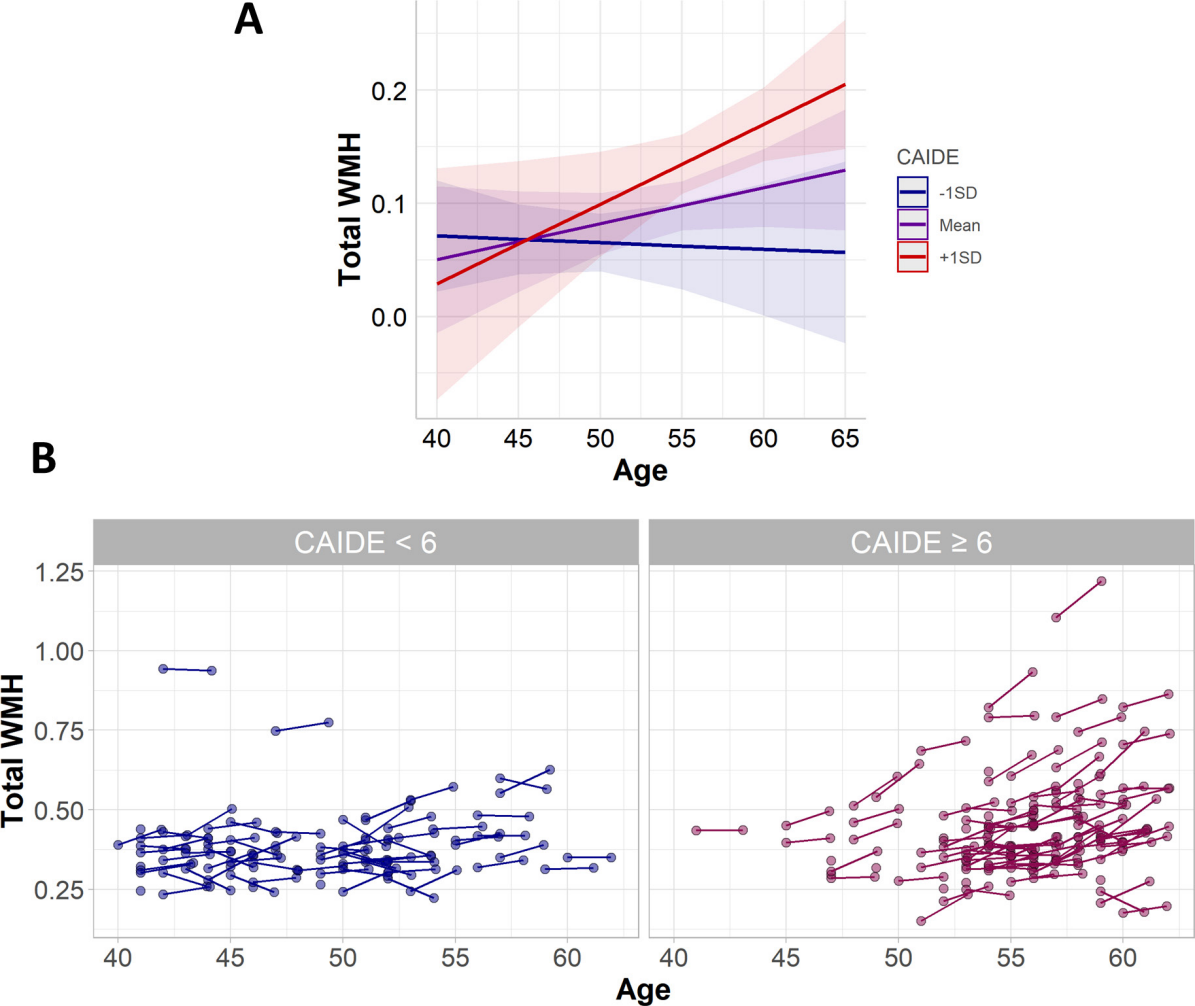
Interaction between age and CAIDE score. (A) Interaction plot of marginal effects. (B) Scatter plot of WMH volume across age in CAIDE <6 and CAIDE ≥6; includes data from both timepoints of all participants (n=185), including those without follow-up measurements (single timepoint). CAIDE, Cardiovascular Risk Factors, Ageing and Dementia score; WMH, white matter hyperintensities.

Using linear mixed-effects modelling, cross-sectional interaction analyses showed that higher baseline CAIDE scores amplified the effect of older age (age at visit) on total WMH (t=2.40, p=0.017), periventricular WMH (t=2.81, p=0.005), global SVD (t=2.08, p=0.039), CAA-SVD (t=2.71, p=0.007), CRP (t=5.94, p<0.001), fibrinogen (t=2.27, p=0.024), but not deep WMH (t=1.75, p=0.081) or hypertensive arteriopathy (t=0.46, p=0.646) (figure 5A). The *CAIDE×age* interaction effect on WMH was confirmed using separate general linear models, whereby associations between age and total WMH was significant in the subset with high (CAIDE ≥6; t=2.50, p=0.013), but not low (CAIDE <6; t=0.15, p=0.882) CAIDE scores (figure 5B).

### Cognition

#### Main effects

Baseline CAIDE scores were associated with poorer performance on reaction time (r_s_=0.16, p=0.031) and the Stroop test (r_s_=−0.26, p<0.001), but were not associated with memory tasks (immediate recall: r_s_=−0.02, p=0.790; narrative recall: r_s_=−0.04, p=0.570). Longitudinally, however, baseline CAIDE scores predicted greater decline in memory (immediate recall: t=−2.34, p=0.021; but not narrative recall: t=−0.31, p=0.754), and were not related to longitudinal changes in reaction time (t=−0.01, p=0.996) or the Stroop test (t=−0.80, p=0.424).

The composite score of hypertensive arteriopathy was associated with longer reaction time (r_s_=0.15, p=0.047), although this association was not significant in relation to global SVD (r_s_=0.13, p=0.068), CAA-SVD (r_s_=0.08, p=0.276) or other individual SVD markers.

#### Interaction effects

Significant *WMH×CAIDE* interactions were observed at baseline in relation to narrative recall (t=−2.93, p=0.004), such that the effect of WMH burden on poorer narrative recall was more pronounced in individuals with higher CAIDE scores.

## Discussion

In our sample of healthy midlife adults, higher CAIDE risk scores were associated cross-sectionally with greater SVD burden (WMH, CMB, lacunes, EPVS, composite SVD scores) and systemic inflammation (CRP, fibrinogen). Longitudinally, higher CAIDE scores predicted greater progression of WMH, but not EPVS, new CMB or inflammation. Moreover, higher CAIDE scores amplified the age-related increase in SVD burden and inflammation, and exacerbated the effect of SVD on cognition.

Cross-sectionally, higher CAIDE scores were associated with greater severity of all SVD markers, namely WMH, EPVS, lacunes, CMB and composite SVD scores. Age and hypertension appeared to contribute the most to SVD risk, with hypertension being selectively associated with SVD distribution characteristic of hypertensive arteriopathy (eg, deep CMB, EPVS in basal ganglia), but not with SVD associated with a CAA-SVD pattern of distribution (lobar CMB, EPVS in centrum semiovale).

Longitudinally, the CAIDE score predicted 2-year progression of WMH volume, but not other biomarkers. The lack of CAIDE associations with other SVD markers may stem from low incidence of new lesions in this healthy cohort of relatively young midlife adults. The association with WMH detectable during a short follow-up period of 2 years highlights its usefulness as an early marker of cerebrovascular alterations in healthy individuals at higher risk of developing dementia (as measured by CAIDE), and its early vulnerability to risk factors. Specifically, older age and APOE4 were the main contributors to WMH progression. This association with APOE4 may be attributed to CAA or Alzheimer’s pathology; while WMH is widely recognised as a marker of SVD, it can also have mixed aetiologies like Wallerian degeneration. Adjusting for all other components, no single item of the CAIDE score emerged as significant predictors of progression in other SVD markers.

Elevated levels of systemic inflammation were also associated with higher CAIDE scores. This is consistent with reports of associations between inflammation and individual CAIDE components, for example, age, hypertension.^34 35^ In our present study, older age and obesity were the most predictive factors of systemic inflammation at baseline, possibly through a mechanistic pathway involving oxidative stress,^34^ while lower education was the main predictor of increasing levels of systemic inflammation over 2 years. Growing literature on the topic suggests that the heightened risk of dementia linked to systemic inflammation may be attributed to the combination of age-related risk factors, oxidative stress, endothelial dysfunction and breakdown of the blood-brain barrier.^27 35 36^

In addition to showing that age and dementia risk (CAIDE score) increased the risk of SVD, our data demonstrated that these effects were synergistic, such that the effect of older age was exacerbated by greater overall dementia risk. This suggests that general dementia risk may confer a *heightened* vulnerability of ageing vessels to vascular injury, atherosclerotic lesions, impaired angiogenesis and dysfunction of the ageing endothelium.^37^ Accordingly, this highlights the importance of considering the differential effects of risk factors at different stages in the adult lifespan. This is supported by earlier studies demonstrating stronger predictive value of risk factors at midlife rather than late-life.^3–6 38^ In particular, the amplification of the effect of age on SVD was observed with respect to CAA-SVD, but not hypertensive arteriopathy. In hypertensive arteriopathy, the effect of the CAIDE score was stable across age span. Conversely, the effects of age and dementia risk on CAA-SVD were synergistic. This could be attributed to the different mechanisms underlying each SVD subtype—while hypertensive arteriopathy is more strongly linked to vascular risk factors and less so with age, CAA-SVD is thought to be caused by the deposition of amyloid-beta in vessel walls which could be exacerbated by age, cerebrovascular disease and APOE4. These results support the notion that distinct aetiologies are involved in the two forms of SVD and suggests that the clinical management of the two subtypes should be considered separately.

In terms of cognition, higher CAIDE scores related to poorer reaction time and executive functioning (deficits characteristic of SVD) at baseline. Longitudinally, baseline CAIDE scores predicted greater decline in memory performance (deficits characteristic of AD). Furthermore, the effect of the CAIDE score on poorer baseline memory was exacerbated in the presence of high WMH burden, while this moderating effect was not observed in relation to reaction time or executive function.

Our present findings demonstrate the potential utility of the CAIDE score as both a *prognostic* and *predictive* marker in the context of cerebral SVD. *Prognostic* markers estimate the likelihood of disease progression in individuals with the disease or a condition of interest. Accordingly, the present data highlight the value of the CAIDE score as a prognostic midlife marker of (1) WMH progression and (2) poorer cognition. *Predictive* markers, on the other hand, identify individuals who are more likely to benefit from different therapeutic approaches. In addition to its ability to identify individuals at greater risk of cerebrovascular disease, analyses of the CAIDE score—both as a composite score and its individual components—suggest that targeting modifiable lifestyle changes could be effective as a potential therapeutic approach to reduce age-related cerebrovascular damage and cerebrovascular-related cognitive impairment, potentially attenuating the detrimental effect of age on cerebrovascular health. While further research would be paramount in establishing the CAIDE score as a prognostic and predictive marker, present findings highlight its potential to inform clinical management and research (eg, clinical trials).

Key strengths of this study include its longitudinal design and comprehensive characterisation of SVD—all four major MRI markers of SVD were assessed, and region-specific burden was quantified for each SVD marker. In addition to global SVD burden,^26^ regional SVD quantification also enabled us to compute composite scores of different SVD subtypes, that is, CAA-SVD and hypertensive arteriopathy.

Another strength of the PREVENT study is the acquisition of thin-slice MRI (eg, 1 mm in T1-weighted scans, 1.2 mm in SWI), improving the detection of smaller lesions and reducing the inherent issue of longitudinal lesion detection affected by different positioning of participants in scanner between different timepoints. Following the STRIVE guidelines,^39^ lacunes tend to be minimally 3 mm in diameter, and were therefore still detectable in our T1-weighted scans, even if participant positioning shifts across timepoints.

Furthermore, our sample of relatively young subclinical midlife adults allowed us to detect early biomarker changes in individuals at risk of developing dementia, thus informing early stage pathological processes. In comparison to similar cohorts of healthy individuals, the prevalence of SVD markers in the PREVENT cohort was slightly higher,^40^ likely due to the oversampling of at-risk individuals, including carriers of the APOE4 allele, a well-established risk factor of SVD (particularly CAA-SVD) and AD. For CMB specifically, higher incidence may be further attributed to more sensitive detection afforded by thin-sliced (1.2 mm; as opposed to thick-sliced) SWI (as opposed to gradient recalled echo (GRE)) scans acquired at 3 T (as opposed to 1.5 T) MRI, which detect up to three times the number of CMB compared with conventional 1.5 T GRE.^41^

Limitations of the study include the lack of data on peripheral diseases, cardiovascular lesions, atheroma or similar lesions, which could contribute to the increase in SVD lesions. As such, we were unable to disentangle the effects on *brain-specific* SVD from systemic vascular disease. Given that systemic vascular disease can manifest as a multisystem disorder affecting the small vessels of the heart, brain and possibly other organs,^42^ the specificity of these associations with risk factors and their effects on dementia risk warrants further investigation using a multisystem approach. Furthermore, our composite score of CAA-SVD did not include all radiological markers of CAA (eg, intracranial haemorrhage, cortical superficial siderosis). Our simplified scoring system of CAA-SVD (and hypertensive arteriopathy) was modelled after an existing rating scale by Staals *et al* 26 for accessibility, such that researchers who have the data to compute this composite global SVD burden score should be able to compute the two composite scores of SVD subtypes, that is, hypertensive arteriopathy and CAA-SVD, assuming that some minimal regional information (eg, deep/lobar) is captured during visual rating. In other words, some degree of exactness in measuring CAA-SVD was sacrificed for the sake of accessibility and comparability to hypertensive arteriopathy and global SVD. Other limitations include the measurement of inflammation using peripheral blood markers (CRP, fibrinogen), which may not be reflective of inflammation in the central nervous system. Further investigations should be conducted with central markers of inflammation within the brain, and with more comprehensive panels of blood inflammatory markers. Deeper examinations of associations between measures of systemic inflammation and neuroinflammation in the brain and consequent effects on cognitive impairment are also warranted. Finally, statistical testing of lacune progression could not be conducted due to the low number of participants who developed new lacunes between baseline and follow-up visits. Future studies with larger, multiethnic cohorts and longer follow-up periods will be crucial to examine the role of vascular risk on disease pathology across the adult lifespan, and in relation to other risk factors and biomarkers. These will be addressed in future PREVENT studies as data become available, and further follow-up visits are completed.

## Conclusion

In our sample of cognitively healthy midlife adults, higher CAIDE risk scores were associated with greater cerebrovascular burden and systemic inflammation at baseline, and predicted the longitudinal progression of WMH. Furthermore, higher CAIDE risk scores appeared to exacerbate age-related cerebrovascular pathology. Findings suggest that lifestyle changes aimed at reducing modifiable risk factors at midlife may be effective in reducing age-related cerebrovascular damage and cerebrovascular-related dementia risk.

## Data Availability

Data are available on reasonable request. Data are available on reasonable request to the principal investigator.
