# Supplementary material for: CAIDE dementia risk score relates to severity and progression of cerebral small vessel disease in healthy midlife adults: the PREVENT-Dementia study
Source: J Neurol Neurosurg Psychiatry. 2022 Feb 8;93(5):481–90. doi: 10.1136/jnnp-2021-327462 (PMC9016254; doi:10.1136/jnnp-2021-327462)
Supplement: Supplementary data [file jnnp-2021-327462supp001.pdf]

SUPPLEMENTARY MATERIALS

CAIDE dementia risk score relates to severity and progression of cerebral small vessel disease in healthy midlife adults: the PREVENT-Dementia study

Participant recruitment

The protocol of the PREVENT-Dementia study has been described in detail previously.[1] Cognitively healthy, midlife participants aged 40 to 59 were recruited through multiple sources. Initially, participants were identified from carers and other unaffected family members on the dementia register database held at West London Mental Health National Health Service (NHS) Trust, which holds information on patients with dementia and cognitive impairment who have consented to be approached for clinical research, and their carers (often offspring). Other participants were recruited from control participants registered with the Join Dementia Research website (https://www.joindementiaresearch.nihr.ac.uk/), or from those who registered their interest through the PREVENT-Dementia website (https://preventdementia.co.uk/) and following public presentations and engagement sessions.

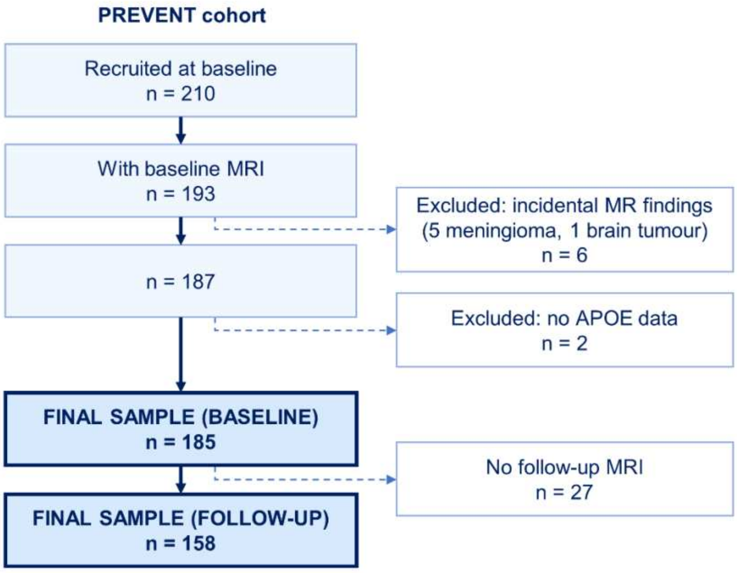

Measures of inflammation

Participants provided fasting blood samples on the morning of their first visit, the same day as clinical and cognitive assessments. Technicians were blinded to all clinical information. Fibrinogen was measured using the Clauss method,[2] by determining the clotting time of diluted plasma after the addition of thrombin using the Dade ® Thrombin Reagent.

Genotyping

Genomic DNA was isolated from whole blood and genotyping was performed in 384 well plates, using the TaqMan polymerase chain reaction (PCR)-based method. The final volume PCR was 5μL using 20ng of genomic DNA, 2.5μL of TaqMan Master Mix, and 0.125 μL of 40× Assay by Design Genotyping Assay Mix, or 0.25μL of 20× Assay on Demand Genotyping Assay. The cycling parameters were 95° for 10 minutes, followed by 40 cycles of denaturation at 92° for 15 seconds and annealing/extension at 60° for one minute. PCR plates were then read on ThermoFisher QuantStudio 12K Flex Real-Time System instrument with QuantStudio 12K Flex Software or TaqMan Genotyper Software v1.3.

MRI acquisition parameters

Three-dimensional T1-weighted MPRAGE parameters were: 160 slices, repetition time (TR)=2300ms, echo time (TE)=2.98ms, flip angle=9°, voxel size=1x1x1mm^3. T2-weighted parameters were: 32 slices, TR=1500ms, TE=80ms, flip angle=150°, voxel-size=0.69x0.69x4mm^3. Fluid-attenuated inversion recovery (FLAIR) parameters were: 27 slices, TR=9000ms, TE=94ms, flip angle=150°, voxel size=0.43x0.43x4mm^3. Susceptibility weighted imaging (SWI) parameters were: 72 slices, TR=28ms, TE=20ms, flip angle=15°, voxel size=0.72x0.72x1.2mm^3.

Quantitative measure of WMH volume

Briefly, SPM8 was used to perform segmentation of T1-weighted images into grey matter (GM), white matter (WM), and cerebrospinal fluid (CSF), based on prior probability maps. Using the GM and WM maps, a brain mask was created and used to perform removal of non-brain matter from the FLAIR images. Initial WMH maps were then obtained using threshold-based segmentation at a threshold of 1.2 times the median pixel intensity of the whole brain, i.e., lesions with pixel intensity more than 1.2 times the median intensity were included in the WMH map. All lesion maps from both baseline and follow-up visits were reviewed by a single experienced rater (A.L.) blinded to all clinical information. Lesion maps obtained from the segmentation procedure were used as starting points for manual WMH delineation. Baseline and follow-up FLAIR images were evaluated side-by-side during delineation to ensure consistency. Binary masks of ventricles underwent four iterations of morphological dilation in MNI space. This dilated ventricular mask was then transformed to subject space to define the boundary between periventricular and deep WMH. Due to the transformation, this is a variable distance accounting for individual brain size, and is approximately 10mm, in line with recommendations.[3]

Semi-quantitative measures of SVD

Periventricular and deep WMH were rated separately: periventricular (0=absent; 1="caps" or pencil-thin lining; 2=smooth "halo"; 3=irregular periventricular signal extending into the deep WM); deep (0=absent; 1=punctate foci; 2=beginning confluence; 3=large confluent areas). CMB were identified on SWI following the Microbleed Anatomical Rating Scale (MARS). Suspected CMB were cross-validated on T1- and T2-weighted scans to exclude CMB 'mimics'. In instances of uncertainty, CMB were labelled as 'possible CMB' – this includes situations whereby CMB cannot be distinguished from vascular flow voids. Such cases of 'possible CMB' were excluded from analysis, and only 'definite CMB' were analysed. Suspected CMB were cross-validated on T1- and T2-weighted scans to exclude CMB 'mimics'. Lacunes and CMB were classified by location as deep (e.g. basal ganglia, thalamus) or lobar (e.g. centrum semiovale) lesions.[4,5] Lacunes and CMB data were dichotomised separately as 'present' (at least one lesion) or 'absent' (no lesions). All lacunes and CMB identified at follow-up were cross-checked against baseline scans to confirm their presence/absence at baseline, and new lesions were flagged. Lacune and CMB progression were defined separately as the presence of any new lesions at follow-up that were confirmed to be absent at baseline.

Neuropsychological measures

Reaction time was measured using a simple reaction time task administered through a touchscreen which records responses and response latencies, and mean reaction time across 12 trials was computed. Executive function was evaluated using the Stroop test in COGNITO, and episodic memory was assessed using a free recall task, and a narrative (story) recall task; further details on the COGNITO battery have been previously described.[6]

Assessing selectiveness of participant drop-out

Firstly, to assess whether participants with complete data differed from those who dropped out (i.e., no follow-up), Mann-Whitney U tests were used to assess whether the two groups differed on baseline age, education, and CAIDE score, while independent chi-square test was used to assess differences in sex.

Participants who dropped out (n=27) were comparable to those with complete data (n=158) in terms of baseline age (Wilcoxon r=-0.10, p=.188), CAIDE score (Wilcoxon r=-.06, p=.379), and sex (X^2=0.09, p=.760), although those who dropped out had fewer years of education (Wilcoxon r=-0.16, p=.028).

REFERENCES

1 Ritchie CW, Ritchie K. The PREVENT study: A prospective cohort study to identify mid-life biomarkers of late-onset Alzheimer's disease. *BMJ Open* 2012;**2**:1–6. doi:10.1136/bmjopen-2012-001893

2 Clauss A. Gerinnungsphysiologische Schnellmethode zur Bestimmung des Fibrinogens. *Acta Haematol* 1957;**17**:237–46. doi:10.1159/000205234

3 Griffanti L, Jenkinson M, Suri S, *et al.* Classification and characterization of periventricular and deep white matter hyperintensities on MRI: A study in older adults. *Neuroimage* 2018;**170**:174–81. doi:10.1016/j.neuroimage.2017.03.024

4 Pasi M, Boulouis G, Fotiadis P, *et al.* Distribution of lacunes in cerebral amyloid angiopathy and hypertensive small vessel disease. *Neurology* 2017;**88**:2162–8. doi:10.1212/WNL.0000000000004007

5 Gregoire SM, Chaudhary UJ, Brown MM, *et al.* The Microbleed Anatomical Rating Scale (MARS): reliability of a tool to map brain microbleeds. *Neurology* 2009;**73**:1759–66. doi:10.1212/WNL.0b013e3181c34a7d

6 Ritchie K, de Roquefeuil G, Ritchie CW, *et al.* COGNITO: Computerized Assessment of Information Processing. *J Psychol Psychother* 2014;**4**:136. doi:10.4172/2161-0487.1000136
